# Genomics-inspired discovery of massiliachelin, an agrochelin epimer from *Massilia* sp. NR 4-1

**DOI:** 10.3762/bjoc.15.128

**Published:** 2019-06-13

**Authors:** Jan Diettrich, Hirokazu Kage, Markus Nett

**Affiliations:** 1 Department of Biochemical and Chemical Engineering, Laboratory of Technical Biology, TU Dortmund University, Emil-Figge-Strasse 66, 44227 Dortmund, Germany

**Keywords:** agrochelin, genome mining, *Massilia*, massiliachelin, siderophore, stereochemistry

## Abstract

A putative siderophore locus was detected in the genome of the violacein-producing bacterium *Massilia* sp. NR 4-1 and predicted to direct the biosynthesis of a molecule that is structurally related to the thiazoline-containing siderophore micacocidin. In order to track this compound, we analyzed the metabolic profiles of *Massilia* cultures grown under different iron concentrations. A compound which was found to be predominantly produced under iron deficiency was subsequently isolated. Its structural characterization by spectroscopic and bioinformatic analyses revealed a previously not known diastereomer of the cytotoxic alkaloid agrochelin. The structure of this natural product, which was named massiliachelin, corresponds to the assembly line encoded by the identified siderophore locus.

## Introduction

In recent years, chemical investigations as well as genomics led to the recognition of a far greater taxonomic diversity of microbes that can produce bioactive compounds [1–3]. The exploration of previously neglected taxa has been demonstrated to bear significant potential for finding new natural products and is thus highly promising from a drug discovery perspective [4–5]. In this study, we analyzed the β-proteobacterium *Massilia* sp. NR 4-1, which had been isolated from a soil sample collected under a nutmeg tree [6]. Although, this strain had already been identified as producer of the antibiotic violacein [6], still not much is known about its secondary metabolism or the chemistry of the genus *Massilia*, in general. Because natural product-competent microorganisms typically synthesize multiple compounds [7], strain NR 4-1 appeared as a promising candidate to find further secondary metabolites. Further incentive for the chemical analysis of this bacterium came from the discovery of novel natural products in the genera *Janthinobacterium* [8] and *Collimonas* [9–10], which are taxonomically closely related to *Massilia*.

Bioinformatic analysis of the 6.36 Mbp-sized genome of strain NR 4-1 using antiSMASH 4.0 [11–12] revealed a total of 16 biosynthesis gene clusters (BGCs), of which one (RS19155-RS19175) could be linked with the production of violacein. Another BGC bears notable similarities to a locus from the plant pathogenic bacterium *Ralstonia solanacearum* GMI1000, which is involved in the biosynthesis of the siderophore micacocidin [13–14]. Differences in the domain organization of the two corresponding biosynthetic assembly lines further indicated that *Massilia* sp. NR 4-1 does not produce micacocidin, but a derivative of this secondary metabolite. The isolation of siderophores from microorganisms is usually straightforward due to their iron-dependent production and complexing properties [15]. Therefore, we decided to initially focus our genome mining efforts on the micacocidin-type cluster in *Massilia* sp. NR 4-1. Here, we report the outcome of this study, which led to the identification of a previously unrecognized agrochelin epimer and, furthermore, unveiled the genetic basis of its biosynthesis.

## Results and Discussion

**Figure 1:**
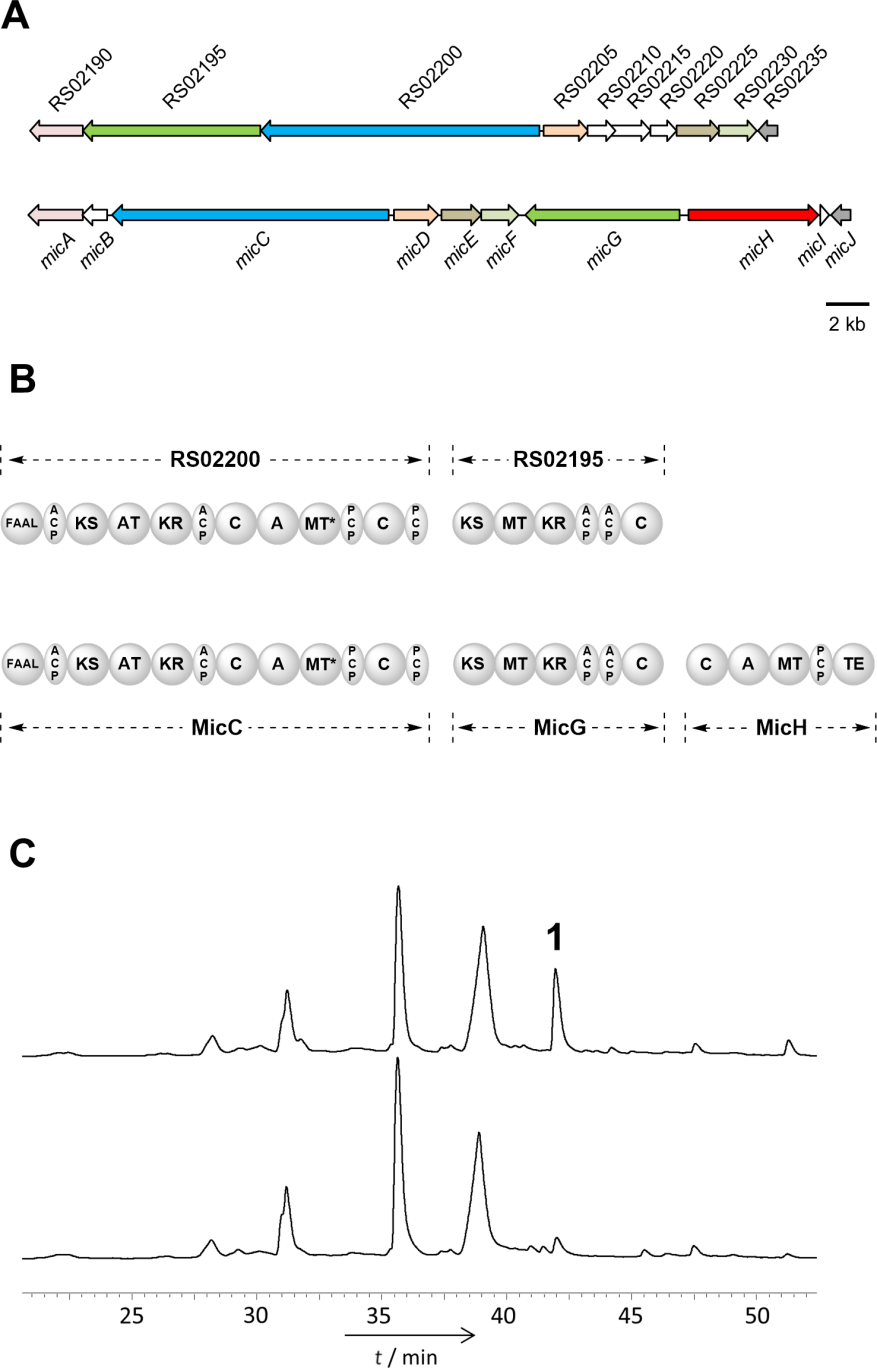
A) Organization of the micacocidin-type gene cluster from *Massilia* sp. NR 4-1 (top) and of the *mic* gene cluster from *R. solanacearum* GMI1000 (bottom). The open reading frames are color-coded according to their predicted function. B) Domain architecture of the massiliachelin (top) and the micacocidin (bottom) assembly lines. Domain notation: FAAL, fatty acyl-AMP ligase; ACP, acyl carrier protein; KS, β-ketoacyl synthase; AT, acyltransferase; KR, ketoreductase; C, condensation; A, adenylation; MT, methyltransferase; PCP, peptidyl carrier protein; TE, thioesterase. The asterisk indicates a methyltransferase-like epimerization domain. C) UV chromatograms of crude culture extracts from *Massilia* sp. NR 4-1 grown under iron-deficient (top) and iron-replete (bottom) conditions. The chromatograms were recorded at a wavelength of 274 nm.

The micacocidin-type BGC from *Massilia* sp. NR 4-1 and its enzymatic assembly line are depicted in Figure 1A and 1B. The gene cluster covers 34.7 kbp of contiguous DNA and includes ten genes (RS02190-RS02235), of which seven have homologs in the *mic* cluster from *R. solanacearum* GMI1000 [13]. A closer inspection of the loci shows a strong conservation of two core biosynthesis genes, namely *micC* and *micG*. On the other hand, the nonribosomal peptide synthetase (NRPS) gene *micH* is missing in the *Massilia* locus. As evidenced by biosynthetic precedence, MicH is responsible for the assembly of a thiazoline ring through condensation of a cysteine residue and subsequent cyclization [16]. The lack of MicH would hence indicate the absence of the corresponding thiazoline motif in the corresponding natural product.

Based upon the assumption that the production of the micacocidin-like compound in *Massilia* sp. NR 4-1 is iron-dependent, we analyzed the HPLC UV profiles of cultures grown under iron-deficient and iron-replete conditions. A comparison of the respective metabolic profiles revealed a distinctive peak, which was massively increased in the extract from the iron-deficient culture (Figure 1C). The corresponding metabolite, which is in the following referred to as massiliachelin (**1**), was subsequently isolated by HPLC.

High-resolution ESIMS of **1** yielded a pseudomolecular ion peak at *m/z* 467.2033 [M + H]^+^, which indicates a molecular formula of C_23_H_34_N_2_O_4_S_2_ and is consistent with eight degrees of unsaturation. NMR measurements confirmed the number of carbon atoms and, furthermore, revealed the presence of 31 non-exchangeable protons (Table 1). Eight carbon atoms of **1** are sp^2^-hybridized according to their chemical shifts. Of these, six could be attributed to a 2,3-substituted phenol moiety (C-1 to C-6), whereas the other two carbons exhibited resonances at 181.3 ppm (C-23) and 182.3 ppm (C-12) characteristic of carbon–heteroatom double bonds. This left two degrees of unsaturation for additional ring structures. HMBC and COSY data indicated that the phenol moiety of **1** bears an *n*-pentyl side chain in *meta*-position to its hydroxy group. Long-range correlations of H-4 and H-6 further established the linkage between C-2 and C-12. In addition, the HMBC experiment detected correlations from H-13 and H-14 to C-12, which, in combination with characteristic chemical shift values [13,17], confirmed the presence of a thiazoline substituent at C-2. Likewise, the adjacent thiazolidine moiety was determined. The ^1^H NMR spectrum of **1** features three methyl singlets, of which one could be assigned to an *N*-methyl group of the thiazolidine by HMBC data. The remaining two methyl groups (CH_3_-21 and CH_3_-22) are connected to C-20. Long-range correlations of their protons also established the position of C-19 and C-23 next to C-20. The hydroxy group at C-19 and the carboxyl group at C-23 were deduced from the carbon chemical shifts of these atoms. In this way, the full planar structure of **1** was elucidated.

**Table 1:** NMR spectroscopic data of **1** in CDCl_3_.

pos.	δ_C_, type	δ_H_, mult. (*J* in Hz)	COSY	HMBC	NOESY

C1	158.8, C				
C2	112.6, C				
C3	144.7, C				
C4	122.2, CH	6.76, d (7.4)	5	1, 2, 3, 6, 7, 12	5, 7a, 7b, 8
C5	135.9, CH	7.33, t (8.2, 7.8)	4, 6	1, 2, 3	4, 6
C6	116.1, CH	7.22, d (8.5)	5	2, 4, 12	5
C7	35.1, CH_2_	a: 2.92, ddd (14.4, 9.8, 6.4) b: 2.76, ddd (14.4, 9.8, 6.3)	7b, 8 7a, 8	2, 3, 4, 8 2, 3, 4, 8	4, 7b, 8, 9 4, 7a, 8, 9
C8	31.8, CH_2_	1.58, m	7a, 7b, 9	3, 7, 9, 10	7a, 7b, 9, 10
C9	31.7, CH_2_	1.33, m	8, 10	7, 11	7a, 7b, 8, 10, 11
C10	22.4, CH_2_	1.33, m	9, 11	8	9, 11
C11	14.0, CH_3_	0.89, t (7.1)	10	9, 10	9, 10
C12	182.3, C				
C13	33.5, CH_2_	a: 3.72, dd (12.1, 9.9) b: 3.46, dd (12.1, 6.4)	13b, 14 13a, 14	12, 14, 15 12, 14, 15	13b, 14 13a, 14, 15
C14	70.7, CH	5.09, td (9.9, 6.4)	13a, 13b, 15	12, 15	13a, 13b, (15), 16
C15	80.4, CH	4.20, d (9.9)	14	13, 14, 16, 17, 18	13b, (14), 18
C16	36.1, CH_2_	3.23, d (7.9)	17	15, 17	14, 17, 19
C17	73.1, CH	3.49, dt (7.9, 2.6)	16, 19	15, 16, 18	16, 18, 19, 22
C18	47.3, CH_3_	2.66, s		15, 17	15, 17, 22
C19	78.6, CH	3.31, d (2.6)	17	16, 20, 21, 22, 23	16, 17, 21, 22
C20	44.3, C				
C21	26.5, CH_3_	1.38, s		19, 20, 22, 23	19
C22	22.1, CH_3_	1.25, s		19, 20, 21, 23	17, 18, 19
C23	181.3, C				

**Figure 2:**
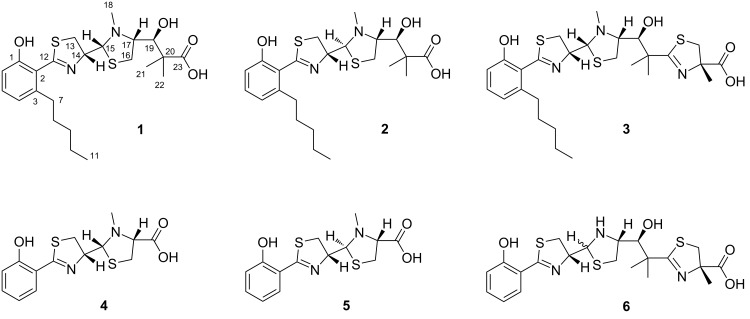
Structures of massiliachelin (**1**), agrochelin (**2**), micacocidin (**3**), pyochelin I (**4**), pyochelin II (**5**), and yersiniabactin (**6**).

According to a literature search, **1** possesses the same chemical constitution as the alkaloid agrochelin (**2**, Figure 2), which was previously reported from a marine bacterium of the genus *Agrobacterium* [18]. However, we also noted some discrepancies in the observed chemical shifts, which suggested that the isolated natural product from *Massilia* sp. is not identical to agrochelin. Instead **1** and **2** are assumed to represent diastereomers. The occurrence of diastereomers is known for natural products, such as pyochelin and yersiniabactin, which are structurally very similar to agrochelin. In both, pyochelin and yersiniabactin, the stereochemical variability was attributed to the thiazoline–thiazolidine motive, which is also present in **1** and **2** [19–22]. In order to deduce the configuration of **1** we used an approach integrating bioinformatics as well as spectroscopy. First, we analyzed whether the isolated natural product possesses a D- or L-configured thiazoline ring. Previous studies had revealed that the D-thiazoline ring in pyochelin is due to an unusual methyltransferase-like epimerization domain in the biosynthesis protein PchE [23]. An analysis of the analogous enzymes in micacocidin [13], yersiniabactin [16], and enantio-pyochelin [24] biosynthesis confirmed that the presence or absence of this feature allows a reliable prediction of the stereochemistry in this position (Table S1, Supporting Information File 1). In every single case, the domain-based configurational prediction matched the experimental assignment [24–28]. By applying this method to the MicC homolog from *Massilia* sp. NR 4-1, the presence of a D-thiazoline ring in **1** can be deduced. Moreover, the thiazolidine ring in **1** must have the stereochemistry at the C2 position derived from L-cysteine. This is because the MicC homolog from *Massilia* sp. NR 4-1 features only a single adenylation domain for the activation of L-cysteine, which corresponds to the micacocidin and yersiniabactin assembly lines [13,16]. An inspection of the ketoreductase (KR) domain in the MicG homolog from *Massilia* sp. NR 4-1 revealed an aspartic acid residue at position 95 and a proline residue at position 144, which are both indicative for the formation of B-type alcohol stereochemistry [29]. The two motifs are also conserved in MicG and HMWP1 from micacocidin and yersiniabactin biosynthesis (Table S2, Supporting Information File 1), which led us to infer that the absolute configuration at C-19 is *S*. It was hence possible to predict the configuration of all stereocenters in **1** except C-15 by bioinformatics. To conclude the stereochemical analysis, we resorted to the NOESY spectrum of **1**. Cañedo and co-workers had previously reported a (14*R*,15*S*,17*R*,19*S*) relative configuration for **2** [18]. As opposed to the NMR analysis of **2**, we detected only a weak NOE correlation between H-14 and H-15. Furthermore, strong NOEs were observed from the *N*-methyl protons (H_3_-18) to both, H-17 and H-15, which is only possible if the latter two protons are *syn*-oriented. We thus propose (14*R*,15*R*,17*R*,19*S*) configuration for **1**. It is noteworthy that the same conclusion can also be drawn from a comparison of the ^1^H NMR data of the thiazoline-thiazolidine moieties in pyochelin and agrochelin (Table S3, Supporting Information File 1).

## Conclusion

In summary, *Massilia* sp. NR 4-1 was found to synthesize an epimer of the alkaloid agrochelin under iron-deficient conditions. The structure of massiliachelin is consistent with the architecture of a biosynthetic assembly line, which is encoded in the genome of this bacterium. Bioinformatic analyses greatly facilitated the stereochemical analysis and also demonstrated the usefulness of computational methods in the configurational assignment of this class of natural products.

## Experimental

### Analytical methods

LC–MS analyses were performed with a Nucleoshell RP18 column (150 × 2.0 mm, Macherey-Nagel) using an Agilent 1260 Infinity LC system combined with a Compact quadrupole-time of flight (Q-TOF) mass spectrometer (Bruker Daltonics). The Q-TOF mass spectrometer was interfaced with an electrospray ionization source. NMR spectra were recorded on a Bruker AV 600 MHz Avance III HD system with chloroform-*d* as solvent and internal standard. The solvent signals were referenced to δ_H_ 7.24 ppm and δ_C_ 77.0 ppm.

### Cultivation and extraction of *Massilia* sp. NR 4-1

Strain NR 4-1 was cultured in three 5 L Simax flasks each containing two liters of R2A medium (0.5 g/L yeast extract, 0.5 g/L proteose peptone, 0.5 g/L casamino acids, 0.5 g/L glucose, 0.5 g/L soluble starch, 0.3 g/L sodium pyruvate, 0.3 g/L K_2_HPO_4_ and 0.05 g/L MgSO_4_·7H_2_O, pH 7.2). The cultures were shaken at 160 rpm and 30 °C. After seven days of cultivation the fermentation broth was extracted three times with ethyl acetate and the solvent was removed in vacuo to give 356 mg of dried extract.

### Isolation of massiliachelin (**1**)

The extract was dissolved in 3 mL methanol and purified by reversed-phase HPLC with a Nucleodur 18 PAH column (250 × 8.0 mm, 3 µm, Macherey Nagel) using a linear gradient of methanol in water supplemented with 0.1% (v/v) trifluoroacetic acid. The gradient conditions were as follows: 10% methanol for 5 min, from 10% to 100% over 35 min, 100% for 10 min, followed by 10% for 10 min. The flow rate was set to 1 mL/min. The elution of compounds was monitored with a diode array detector. In total, 12.0 mg of **1** were isolated.

### Massiliachelin (**1**)

pale yellow solid; UV (MeOH) λ_max_ (log ε): 356 (3.06), 280 (4.18), 228 (4.64) nm; ^1^H and ^13^C NMR, Table 1; HRESIMS *m/z*: [M + H]^+^, calcd for C_23_H_35_N_2_O_4_S_2_, 467.2033; found, 467.2033.

## Supporting Information

File 1 Additional tables and copies of NMR spectra.

## References

[1] Masschelein J, Jenner M, Challis G L (2017). Nat Prod Rep.

[2] Challinor V L, Bode H B (2015). Ann N Y Acad Sci.

[3] Molloy E M, Hertweck C (2017). Curr Opin Microbiol.

[4] Ling L L, Schneider T, Peoples A J, Spoering A L, Engels I, Conlon B P, Mueller A, Schäberle T F, Hughes D E, Epstein S (2015). Nature.

[5] Lincke T, Behnken S, Ishida K, Roth M, Hertweck C (2010). Angew Chem, Int Ed.

[6] Myeong N R, Seong H J, Kim H-J, Sul W J (2016). J Biotechnol.

[7] Bode H B, Bethe B, Höfs R, Zeeck A (2002). ChemBioChem.

[8] Graupner K, Scherlach K, Bretschneider T, Lackner G, Roth M, Gross H, Hertweck C (2012). Angew Chem, Int Ed.

[9] Fritsche K, van den Berg M, de Boer W, van Beek T A, Raaijmakers J M, van Veen J A, Leveau J H J (2014). Environ Microbiol.

[10] Kai K, Sogame M, Sakurai F, Nasu N, Fujita M (2018). Org Lett.

[11] Blin K, Pascal Andreu V, de los Santos E L C, Del Carratore F, Lee S Y, Medema M H, Weber T (2019). Nucleic Acids Res.

[12] Blin K, Wolf T, Chevrette M G, Lu X, Schwalen C J, Kautsar S A, Suarez Duran H G, de los Santos E L C, Kim H U, Nave M (2017). Nucleic Acids Res.

[13] Kreutzer M F, Kage H, Gebhardt P, Wackler B, Saluz H P, Hoffmeister D, Nett M (2011). Appl Environ Microbiol.

[14] Kage H, Kreutzer M F, Wackler B, Hoffmeister D, Nett M (2013). Chem Biol.

[15] Kreutzer M F, Nett M (2012). Org Biomol Chem.

[16] Miller D A, Luo L, Hillson N, Keating T A, Walsh C T (2002). Chem Biol.

[17] Souto A, Montaos M A, Rivas A J, Balado M, Osorio C R, Rodríguez J, Lemos M L, Jiménez C (2012). Eur J Org Chem.

[18] Cañedo L M, de la Fuente J A, Gesto C, Ferreiro M J, Jiménez C, Riguera R (1999). Tetrahedron Lett.

[19] Ankenbauer R G, Cox C D (1988). J Bacteriol.

[20] Rinehart K L, Staley A L, Wilson S R, Ankenbauer R G, Cox C D (1995). J Org Chem.

[21] Schlegel K, Taraz K, Budzikiewicz H (2004). BioMetals.

[22] Drechsel H, Stephan H, Lotz R, Haag H, Zähner H, Hantke K, Jung G (1995). Liebigs Ann.

[23] Patel H M, Tao J, Walsh C T (2003). Biochemistry.

[24] Youard Z A, Mislin G L A, Majcherczyk P A, Schalk I J, Reimmann C (2007). J Biol Chem.

[25] Kobayashi S, Nakai H, Ikenishi Y, Sun W-Y, Ozaki M, Hayase Y, Takeda R (1998). J Antibiot.

[26] Ino A, Hasegawa Y, Murabayashi A (1999). Tetrahedron.

[27] Ino A, Murabayashi A (2001). Tetrahedron.

[28] Miller M C, Parkin S, Fetherston J D, Perry R D, DeMoll E (2006). J Inorg Biochem.

[29] Caffrey P (2003). ChemBioChem.

