# Supplementary material for: Genomics-inspired discovery of massiliachelin, an agrochelin epimer from Massilia sp. NR 4-1
Source: Beilstein J Org Chem. 2019 Jun 13;15:1298–303. doi: 10.3762/bjoc.15.128 (PMC6604713; doi:10.3762/bjoc.15.128)
Supplement: File 1 — Additional tables and copies of NMR spectra. [file Beilstein_J_Org_Chem-15-1298-s001.pdf]

BEILSTEIN JOURNAL OF ORGANIC CHEMISTRY

Supporting Information

for

Genomics-inspired discovery of massiliachelin, an agrochelin epimer from *Massilia* sp. NR 4-1

Jan Diettrich, Hirokazu Kage and Markus Nett

Beilstein J. Org. Chem. **2019,** *15,* 1298–1303. doi:10.3762/bjoc.15.128

Additional tables and copies of NMR spectra

License and Terms: This is a supporting information file under the terms of the Creative Commons Attribution License (http://creativecommons.org/licenses/by/4.0). Please note that the reuse, redistribution and reproduction in particular requires that the authors and source are credited.
The license is subject to the *Beilstein Journal of Organic Chemistry* terms and conditions: (https://www.beilstein-journals.org/bjoc)

Table of contents

Table S1: Epimerization domains in pyochelin-type synthetases and configuration of the generated thiazoline ring. S2

Table S2: Alignment of the residue 88–103 and 134–148 regions of the ketoreductase (KR) domains in MicG-like proteins S2

Table S3: Stereochemistry of siderophores with a thiazoline-thiazolidine motif S2

Figure S1: ^{1}H NMR spectrum of massiliachelin (**1**) in chloroform-*d* S3

Figure S2: ^{1}H-decoupled ^{13}C NMR spectrum of massiliachelin (**1**) in chloroform-*d* S4

Figure S3: ^{1}H,^{1}H COSY spectrum of massiliachelin (**1**) in chloroform-*d* S5

Figure S4: ^{1}H,^{13}C HSQC spectrum of massiliachelin (**1**) in chloroform-*d* S6

Figure S5: ^{1}H,^{13}C HMBC spectrum of massiliachelin (**1**) in chloroform-*d* S7

Figure S6: ^{1}H,^{1}H NOESY spectrum of massiliachelin (**1**) in chloroform-*d* S8

Figure S7: HRESIMS spectrum of massiliachelin (**1**) S9

Table S1: Epimerization domains in pyochelin-type synthetases and configuration of the generated thiazoline ring.

	Protein accession # of PchE homolog	Methyltransferase-like epimerization domain in PchE homolog	Configuration of thiazoline ring (proposed)
Pyochelin	AAD55800	Yes	D
Enantio-pyochelin	ABW70809	No	L
Yersiniabactin	P48633	Yes	D
Micacocidin	CAD15508	Yes	D
Massiliachelin (**1**)	AKU20507	Yes	(D)

Table S2: Alignment of the residue 88–103 and 134–148 regions of the ketoreductase (KR) domains in MicG from *Ralstonia solanacearum* GMI1000, HMWP1 from *Yersinia pestis* KIM6+, and ACZ_RS02195 from *Massilia* sp. NR 4-1.

	Protein accession #	88–103 region	134–149 region	KR-type	Configuration (proposed)
MicG	CAD15512	HLAGIVR**D**APLAAADWR	FSSAASACGA**P**GQGAH	B	*S*
HMWP1	AAC69588	HAAGVLA**D**APLQELDDH	YSSAAATLGA**P**GQSAH	B	*S*
RS02195	WP_050407226	HLAAVVR**D**ATLAAINTE	FSSAATAFGA**P**GQGAY	B	(*S*)

Table S3: Stereochemistry of siderophores with a thiazoline-thiazolidine motif.

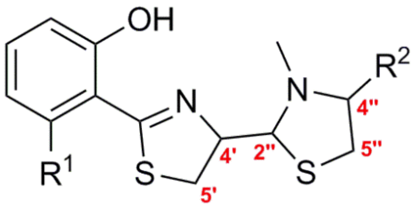

Natural product	Configuration	δ(H-4’), m (*J* in Hz) measured in $CDCl_3$	Reference in main manuscript
Pyochelin I	4’*R*, 2’’*R*, 4’’*R*	5.08, td (8.8, 5.0)	[19]
Yersiniabactin I	4’*R*, 2’’*R*, 4’’*R*	5.15, td (9.2, 4.8)	[26]
Massiliachelin (**1**)	4’*R*, 2’’*R*, 4’’*R*	5.09, td (9.9, 6.4)	This study
Neopyochelin I	4’*S*, 2’’S, 4’’*R*	5.12, td (9.1, 4.6)	[19]
Pyochelin II	4’*R*, 2’’*S*, 4’’*R*	4.92, q (8.2)	[19]
Yersiniabactin II	4’*R*, 2’’*S*, 4’’*R*	4.78, q (n.d.)	[21]
Agrochelin (**2**)	4’*R*, 2’’*S*, 4’’*R*	4.72, q (8.7)	[17]
Neopyochelin II	4’*S*, 2’’*R*, 4’’*R*	4.79, q (8.0)	[19]

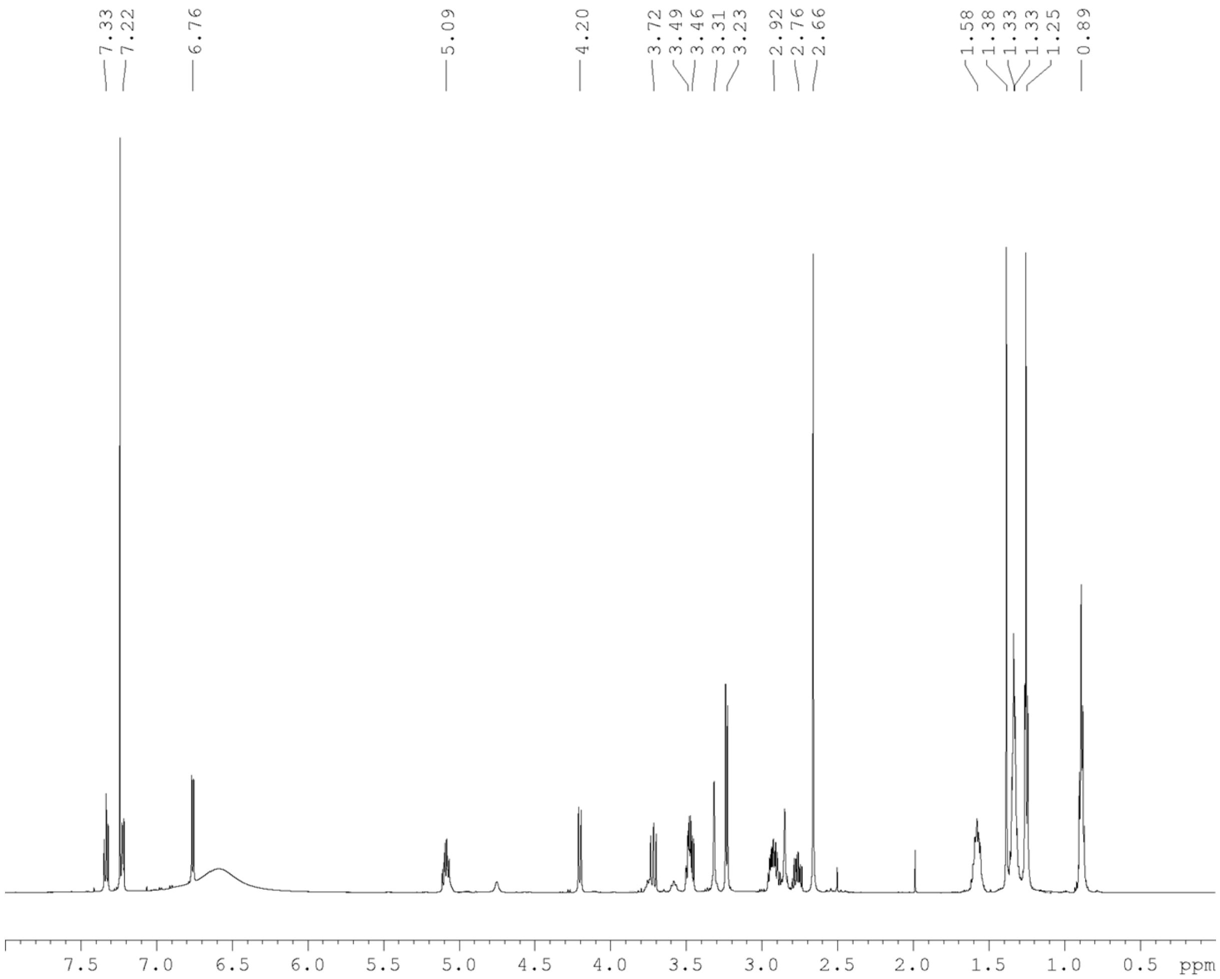

Figure S1: ^{1}H NMR spectrum of massiliachelin (**1**) in chloroform-*d*.

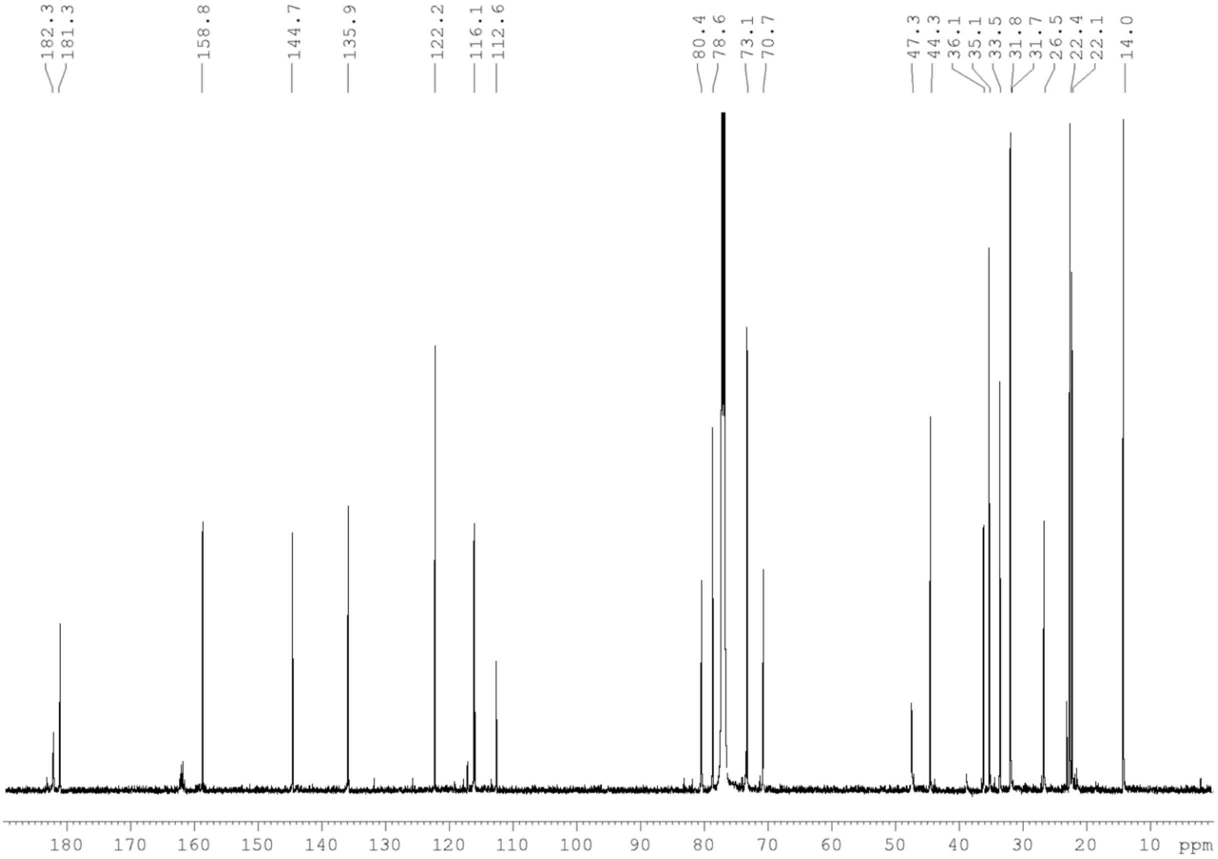

Figure S2: ^{1}H-decoupled ^{13}C NMR spectrum of massiliachelin (**1**) in chloroform-*d*.

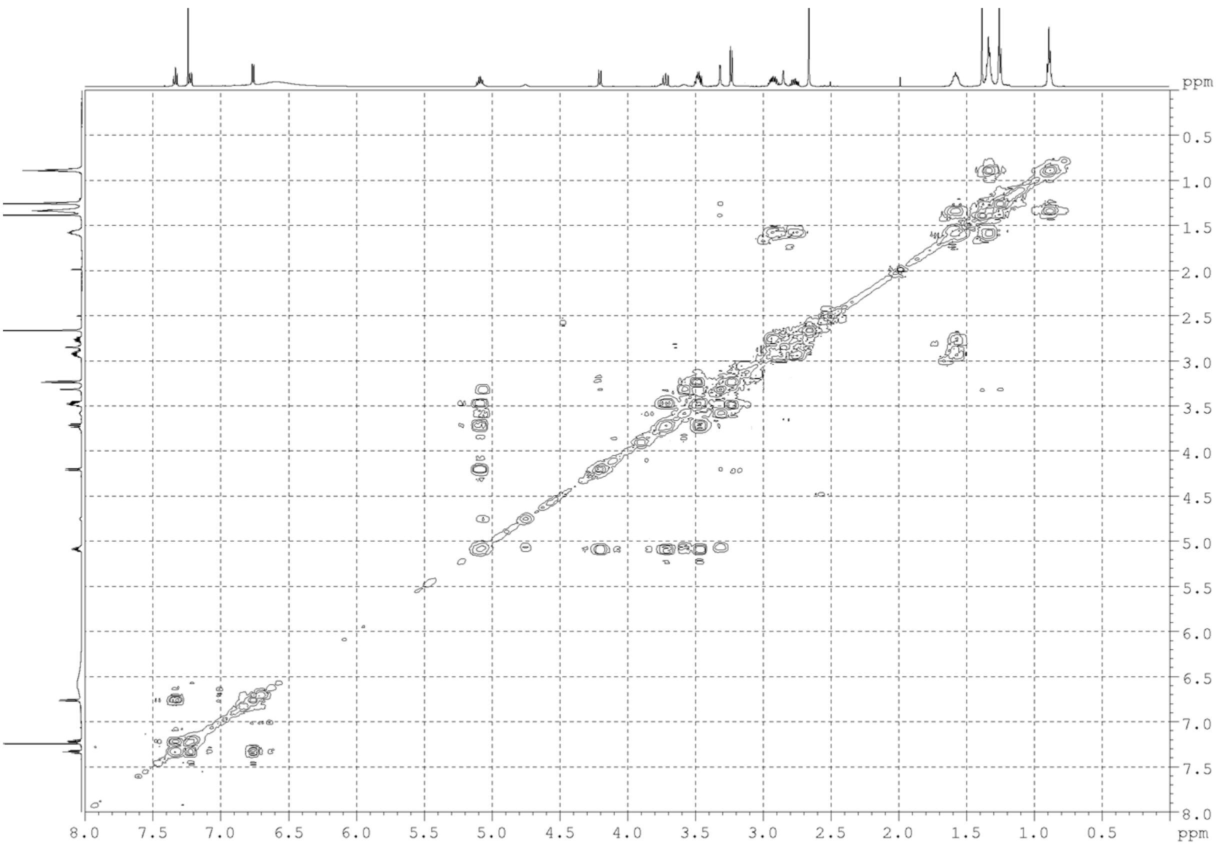

Figure S3: $^{1}H,^{1}H$ COSY spectrum of massiliachelin (**1**) in chloroform-*d*.

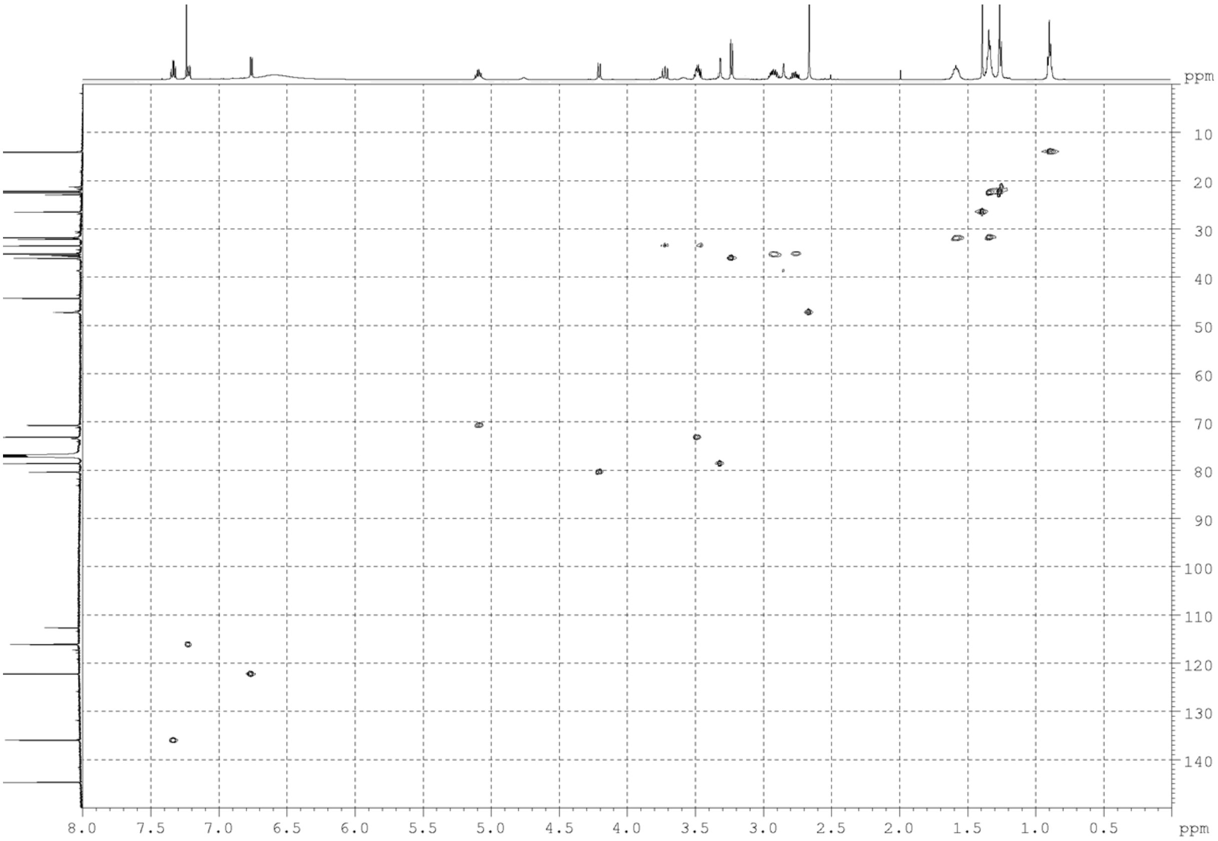

Figure S4: ^{1}H,^{13}C HSQC spectrum of massiliachelin (**1**) in chloroform-*d*.

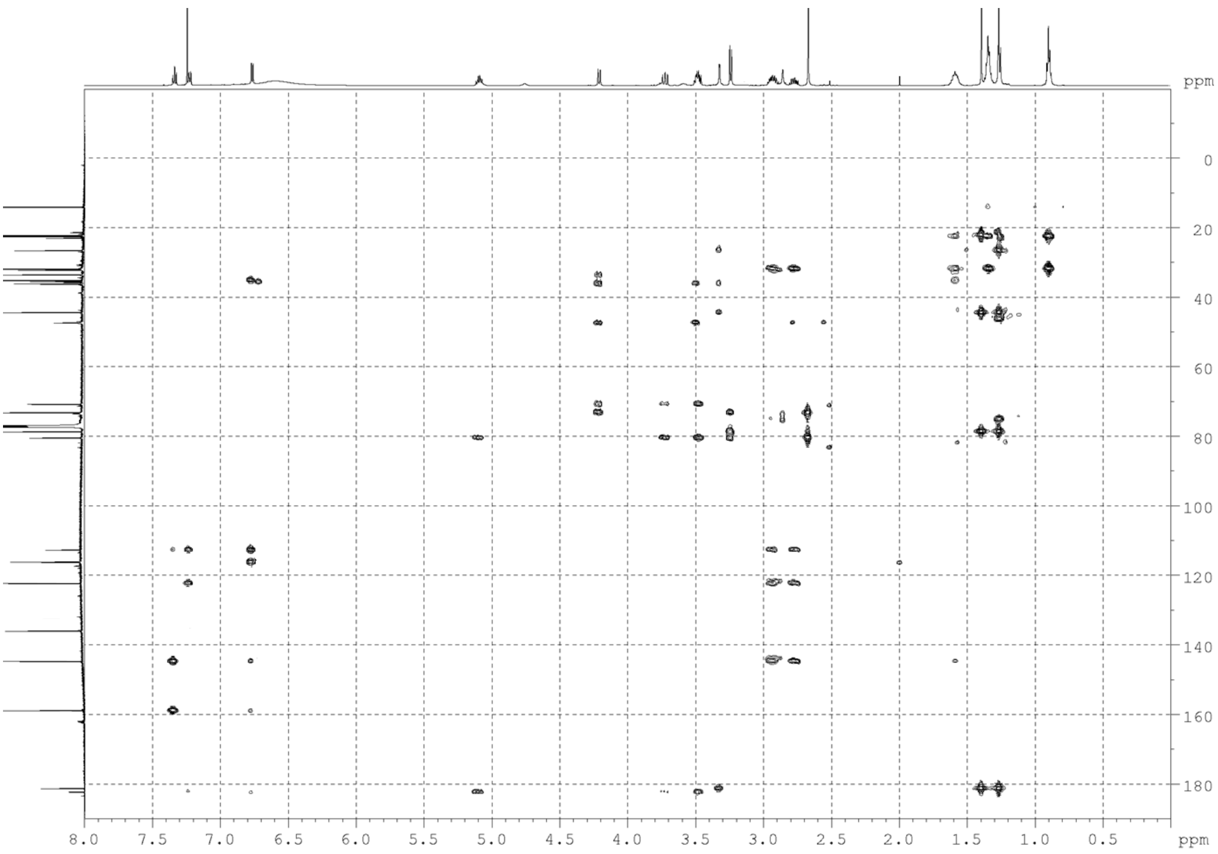

Figure S5: $^{1}H,^{13}C$ HMBC spectrum of massiliachelin (**1**) in chloroform-*d*.

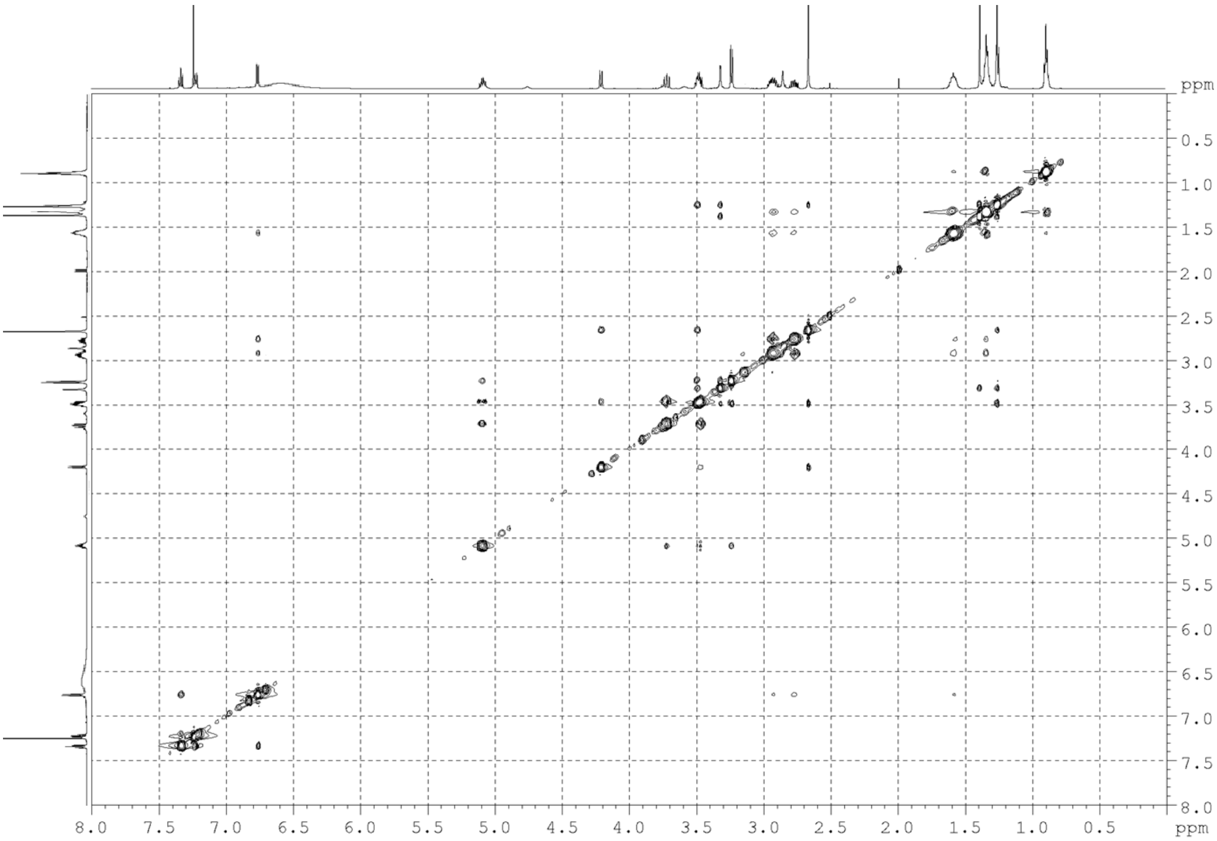

Figure S6: $^{1}H,^{1}H$ NOESY spectrum of massiliachelin (**1**) in chloroform-*d*.

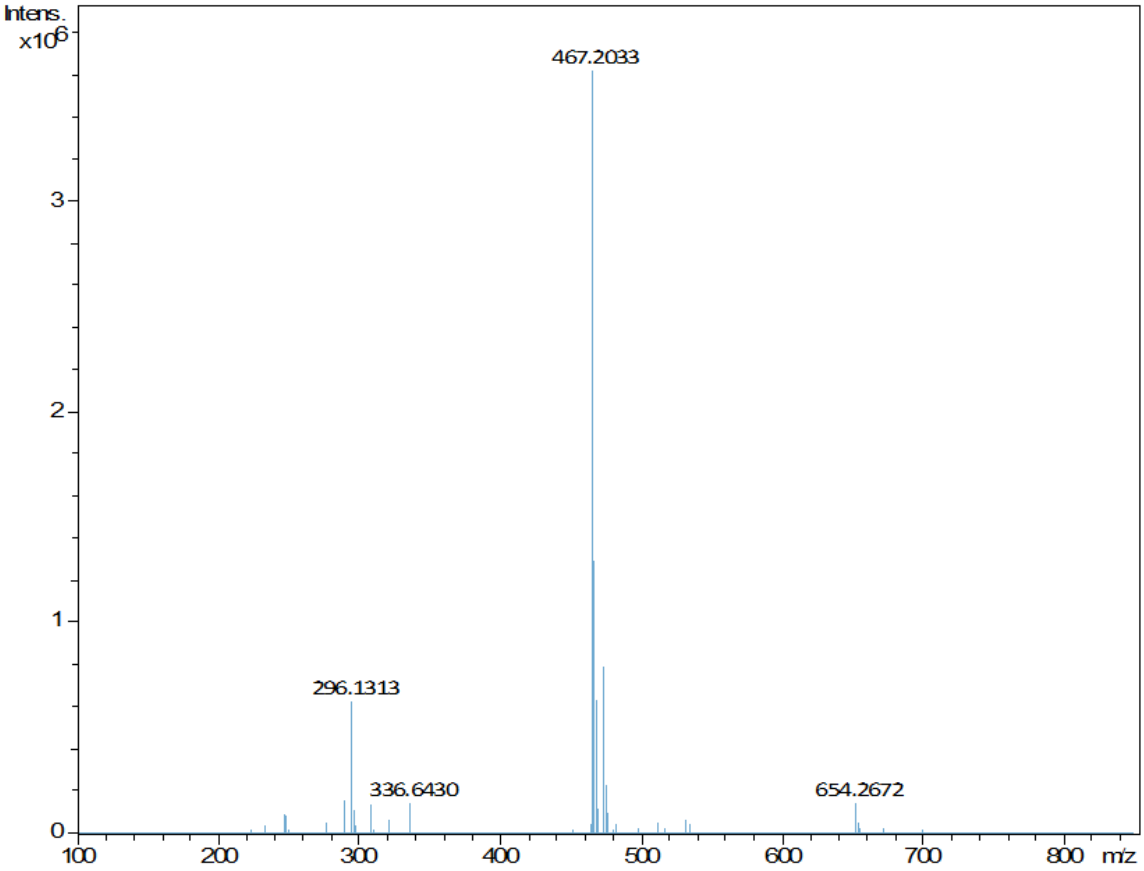

Figure S7: HRESIMS spectrum of massiliachelin (**1**).
